# Retraction: Knockdown of TMEM14A expression by RNAi inhibits the proliferation and invasion of human ovarian cancer cells

**DOI:** 10.1042/BSR-2015-0258_RET

**Published:** 2023-04-21

**Keywords:** TMEM14A, cell cycle, metastasis, ovarian cancer

This Retraction follows an Expression of Concern relating to this article previously published by Portland Press. This article is being retracted from *Bioscience Reports*, at the request of the Editor-in-Chief and the Editorial Board, following receipt of a notification from a reader alerting the Editorial Board to multiple western blots within the article that also seem to appear in other papers by unrelated authors. Specifically, these include the following:

- The Figure 5B Smad2 bands, which seem to appear as the Figure 5A STAT3 bands from Liu et al. 2016 (doi: 10.1038/srep33676);
- the Figure 4C PCNA bands, which seem to appear as the Figure 4B B-catenin bands from Zhu et al. 2016 (doi: 10.1038/srep19070); and the Figure 4C MMP-2 bands, which seem to appear as the Figure 4B B-catenin bands from the same paper by Zhu et al. (2016)
- The Figure 4C MMP-2 bands, which seem to appear as the Figure 2C HDAC1 bands from Wang et al. 2017 (doi: 10.18632/oncotarget.18227)
- The Figure 4C MMP-9 bands, which seem to appear as the Figure 6D VCAM1 bands (vertically flipped) from Cheng et al. 2015 (doi: 10.1186/s13046-015-0193-y)
- The Figure 4D Cyclin E bands, which seem to appear as the Figure 4F CCL3 bands from Wang et al. 2015 (doi: 10.3892/or.2015.4112)

The authors have been contacted with regards to the retraction and have not responded to the Journal's queries or the concerns raised. Given the extent of the issues raised, the Editorial Board stand by the decision to retract the article.

